# Supplementary material for: A Bio-Indicator Pilot Study Screening Selected Heavy Metals in Female Hair, Nails, and Serum from Lifestyle Cosmetic, Canned Food, and Manufactured Drink Choices
Source: Molecules. 2023 Jul 22;28(14):5582. doi: 10.3390/molecules28145582 (PMC10386365; doi:10.3390/molecules28145582)
Supplement: Supplementary file 1 [file molecules-28-05582-s001.zip › molecules-2515995-supplementary.pdf]

Table S1. Protein, Hormone, Gene, and their Receptors used in AutoDock analysis

Target (Protein, Hormone, Gene, and their Receptors) from PDB (3D Structures)				PDB Information
Cholinesterase	Target Protein	1P0P	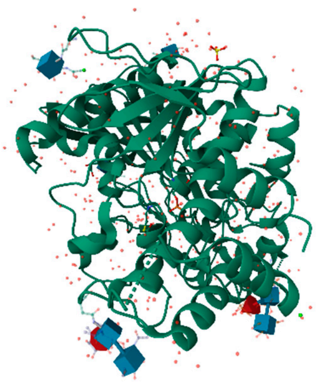	1P0P<br><br>Crystal structure of soman-aged human butyryl cholinesterase in complex with the substrate analog butyrylthiocholine<br><br>• **PDB DOI:** https://doi.org/10.2210/pdb1P0P/pdb<br>• **Classification: HYDROLASE**<br>• **Organism(s):** Homo sapiens<br>• **Expression System:** Cricetulus griseus<br>• **Mutation(s):** Yes<br>• **Deposited:** 2003-04-10 **Released:** 2003-08-05<br>• **Deposition Author(s):** Nicolet, Y., Lockridge, O., Masson, P., Fontecilla-Camps, J.C., Nachon, F.<br><br>**Experimental Data Snapshot**<br><br>• **Method:** X-RAY DIFFRACTION<br>• **Resolution:** 2.30 Å<br>• **R-Value Free:** 0.242<br>• **R-Value Work:** 0.199<br>• **R-Value Observed:** 0.199

	Target<br>Receptor	6EP4	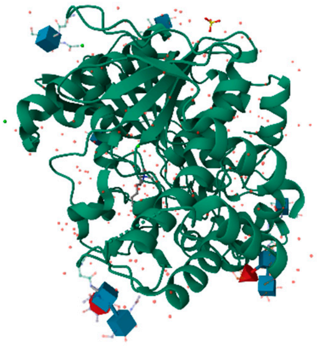	6EP4<br><br>Human butyrylcholinesterase in complex with decamethonium<br><br>• **PDB DOI:** https://doi.org/10.2210/pdb6EP4/pdb<br>• **Classification: HYDROLASE**<br>• **Organism(s):** Homo sapiens<br>• **Expression System:** Cricetulus griseus<br>• **Mutation(s):** Yes<br>• **Deposited:** 2017-10-10 **Released:** 2017-12-13<br>• **Deposition Author(s):** Nachon, F., Brazzolotto, X., Wandhammer, M., Trovaslet-Leroy, M., Rosenberry, T.L., Macdonald, I.R., Darvesh, S.<br>• **Funding Organization(s):** French Ministry of Defense<br><br>**Experimental Data Snapshot**<br><br>• **Method:** X-RAY DIFFRACTION<br>• **Resolution:** 2.30 Å<br>• **R-Value Free:** 0.217<br>• **R-Value Work:** 0.179<br>• **R-Value Observed:** 0.181

P53	Target Protein	7VOU	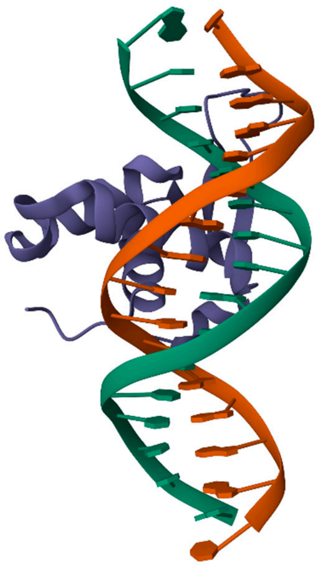	7VOU<br>*The crystal structure of human forkhead box protein in complex with DNA 1*<br>• **PDB DOI:** https://doi.org/10.2210/pdb7VOU/pdb<br>• **NDB:** 7VOU<br>• **Classification:** **TRANSCRIPTION/DNA**<br>• **Organism(s):** Homo sapiens<br>• **Expression System:** Escherichia coli<br>• **Mutation(s):** No<br>• **Deposited:** 2021-10-14 **Released:** 2022-08-17<br>• **Deposition Author(s):** Choi, Y., Yoon, H.J., Lee, H.H.<br>• **Funding Organization(s):** National Research Foundation (NRF, Korea)<br>**Experimental Data Snapshot**<br>• **Method:** X-RAY DIFFRACTION<br>• **Resolution:** 3.10 Å<br>• **R-Value Free:** 0.280<br>• **R-Value Work:** 0.221<br>• **R-Value Observed:** 0.227
		2LY4	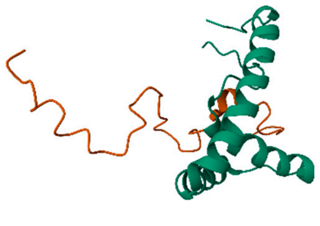	2LY4<br>*HMGB1-facilitated p53 DNA binding occurs via HMG-box/p53 transactivation domain interaction and is regulated by the acidic tail*<br>• **PDB DOI:** https://doi.org/10.2210/pdb2LY4/pdb<br>• **BMRB:** 18709<br>• **Classification:** **NUCLEAR PROTEIN/ANTITUMOUR PROTEIN**<br>• **Organism(s):** Homo sapiens<br>• **Expression System:** Escherichia coli<br>• **Mutation(s):** No<br>• **Deposited:** 2012-09-12 **Released:** 2012-10-31<br>• **Deposition Author(s):** Rowell, J.P., Simpson, K.L., Stott, K., Watson, M., Thomas, J.O.<br>**Experimental Data Snapshot**<br>• **Method:** SOLUTION NMR<br>• **Conformers Calculated:** 100<br>• **Conformers Submitted:** 10<br>• **Selection Criteria:** structures with the lowest energy

		2JTX	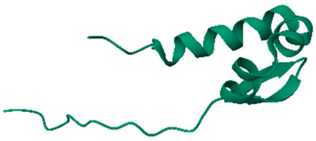	2JTX<br><br>*NMR structure of the TFIIE-alpha carboxyl terminus*<br><br>• **PDB DOI:** https://doi.org/10.2210/pdb2JTX/pdb<br>• **Classification: TRANSCRIPTION**<br>• **Organism(s):** Homo sapiens<br>• **Expression System:** Escherichia coli<br>• **Mutation(s):** No<br>• **Deposited:** 2007-08-08 **Released:** 2007-12-11<br>• **Deposition Author(s):** Di Lello, P., Omichinski, J.G.<br><br>**Experimental Data Snapshot**<br><br>• **Method:** SOLUTION NMR<br>• **Conformers Calculated:** 50<br>• **Conformers Submitted:** 20<br>• **Selection Criteria:** structures with the lowest energy
	Target Receptor	6VTH	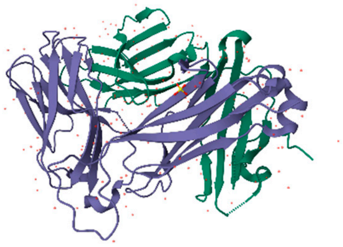	6VTH<br><br>*p53-specific T cell receptor*<br><br>• **PDB DOI:** https://doi.org/10.2210/pdb6VTH/pdb<br>• **Classification: IMMUNE SYSTEM**<br>• **Organism(s):** Homo sapiens<br>• **Expression System:** Escherichia coli<br>• **Mutation(s):** No<br>• **Deposited:** 2020-02-12 **Released:** 2020-06-17<br>• **Deposition Author(s):** Wu, D., Gallagher, D.T., Gowthaman, R., Pierce, B.G., Mariuzza, R.A.<br>• **Funding Organization(s):** National Institutes of Health/National Institute of General Medical Sciences (NIH/NIGMS)<br><br>**Experimental Data Snapshot**<br><br>• **Method:** X-RAY DIFFRACTION<br>• **Resolution:** 2.36 Å<br>• **R-Value Free:** 0.264<br>• **R-Value Work:** 0.211<br>• **R-Value Observed:** 0.214

Dopamine	Target Protein	5PAH	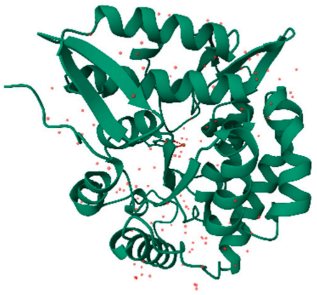	5PAH<br><br>*HUMAN PHENYLALANINE HYDROXYLASE CATALYTIC DOMAIN DIMER WITH BOUND DOPAMINE INHIBITOR*<br><br>• **PDB DOI:** https://doi.org/10.2210/pdb5PAH/pdb<br>• **Classification:** **MONOOXYGENASE**<br>• **Organism(s):** Homo sapiens<br>• **Expression System:** Escherichia coli<br>• **Mutation(s):** No<br>• **Deposited:** 1998-08-20 **Released:** 1999-04-27<br>• **Deposition Author(s):** Erlandsen, H., Flatmark, T., Stevens, R.C.<br><br>**Experimental Data Snapshot**<br><br>• **Method:** X-RAY DIFFRACTION<br>• **Resolution:** 2.10 Å<br>• **R-Value Free:** 0.200<br>• **R-Value Work:** 0.163<br>• **R-Value Observed:** 0.163
	Target Receptor	3PBL	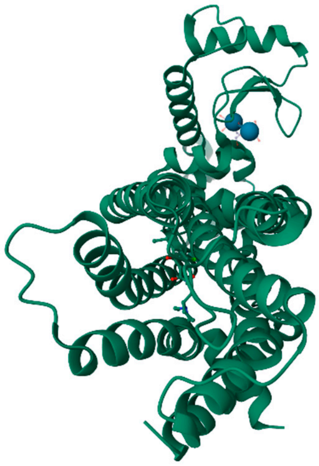	3PBL<br><br>*Structure of the human dopamine D3 receptor in complex with eticlopride*<br><br>• **PDB DOI:** https://doi.org/10.2210/pdb3PBL/pdb<br>• **Classification:** **HYDROLASE/HYDROLASE INHIBITOR**<br>• **Organism(s):** Homo sapiens, Tequatrovirus T4<br>• **Expression System:** Spodoptera frugiperda<br>• **Mutation(s):** Yes<br>• **Membrane Protein:** Yes<br>• **Deposited:** 2010-10-20 **Released:** 2010-11-03<br>• **Deposition Author(s):** Chien, E.Y.T., Liu, W., Han, G.W., Katritch, V., Zhao, Q., Cherezov, V., Stevens, R.C., Accelerated Technologies Center for Gene to 3D Structure (ATCG3D), GPCR Network (GPCR)<br><br>**Experimental Data Snapshot**<br><br>• **Method:** X-RAY DIFFRACTION<br>• **Resolution:** 2.89 Å<br>• **R-Value Free:** 0.272<br>• **R-Value Work:** 0.243<br>• **R-Value Observed:** 0.245

Estrogen	Target Protein	1FDW	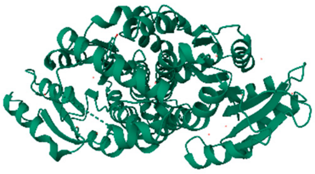	1FDW<br>*HUMAN 17-BETA-HYDROXYSTEROID-DEHYDROGENASE TYPE 1 MUTANT H221Q COMPLEXED WITH ESTRADIOL*<br>• **PDB DOI:** https://doi.org/10.2210/pdb1FDW/pdb<br>• **Classification: DEHYDROGENASE**<br>• **Organism(s):** Homo sapiens<br>• **Expression System:** Spodoptera frugiperda<br>• **Mutation(s):** Yes<br>• **Deposited:** 1998-01-16 **Released:** 1998-05-27<br>• **Deposition Author(s):** Mazza, C., Breton, R., Housset, D., Fontecilla-Camps, J.-C.<br>**Experimental Data Snapshot**<br>• **Method:** X-RAY DIFFRACTION<br>• **Resolution:** 2.70 Å<br>• **R-Value Free:** 0.263<br>• **R-Value Work:** 0.178
	Target Receptor	1L2J	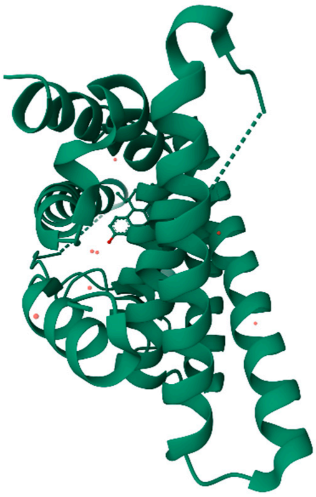	1L2J<br>*Human Estrogen Receptor beta Ligand-binding Domain in Complex with (R,R)-5,11-cis-diethyl-5,6,11,12-tetrahydrochrysene-2,8-diol*<br>• **PDB DOI:** https://doi.org/10.2210/pdb1L2J/pdb<br>• **Classification: transcription receptor**<br>• **Organism(s):** Homo sapiens<br>• **Expression System:** Escherichia coli<br>• **Mutation(s):** No<br>• **Deposited:** 2002-02-21 **Released:** 2002-05-01<br>• **Deposition Author(s):** Shiau, A.K., Barstad, D., Radek, J.T., Meyers, M.J., Nettles, K.W., Katzenellenbogen, B.S., Katzenellenbogen, J.A., Agard, D.A., Greene, G.L.<br>**Experimental Data Snapshot**<br>• **Method:** X-RAY DIFFRACTION<br>• **Resolution:** 2.95 Å<br>• **R-Value Free:** 0.299<br>• **R-Value Work:** 0.259

		4J26	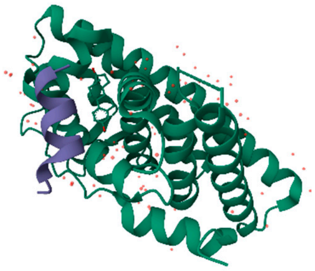	4J26<br>*Estrogen Receptor in complex with proline-flanked LXXLL peptides*<br>• **PDB DOI:** https://doi.org/10.2210/pdb4J26/pdb<br>• **Classification:** **HORMONE RECEPTOR/PEPTIDE**<br>• **Organism(s):** Homo sapiens<br>• **Expression System:** Escherichia coli<br>• **Mutation(s):** No<br>• **Deposited:** 2013-02-04 **Released:** 2013-03-13<br>• **Deposition Author(s):** Fuchs, S., Nguyen, H.D., Phan, T., Burton, M., Nieto, L., de Vries-van Leeuwen, I., Schmidt, A., Goodarzifard, M., Agten, S., Rose, R., Ottmann, C., Milroy, L.G., Brunsveld, L.<br>**Experimental Data Snapshot**<br>• **Method:** X-RAY DIFFRACTION<br>• **Resolution:** 2.30 Å<br>• **R-Value Free:** 0.242<br>• **R-Value Work:** 0.203<br>• **R-Value Observed:** 0.205
		4J24	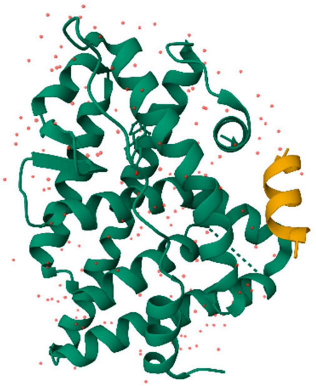	4J24<br>*Estrogen Receptor in complex with proline-flanked LXXLL peptides*<br>• **PDB DOI:** https://doi.org/10.2210/pdb4J24/pdb<br>• **Classification:** **HORMONE RECEPTOR/PEPTIDE**<br>• **Organism(s):** Homo sapiens<br>• **Expression System:** Escherichia coli<br>• **Mutation(s):** No<br>• **Deposited:** 2013-02-04 **Released:** 2013-03-13<br>• **Deposition Author(s):** Fuchs, S., Nguyen, H.D., Phan, T., Burton, M., Nieto, L., de Vries-van Leeuwen, I., Schmidt, A., Goodarzifard, M., Agten, S., Rose, R., Ottmann, C., Milroy, L.G., Brunsveld, L.<br>**Experimental Data Snapshot**<br>• **Method:** X-RAY DIFFRACTION<br>• **Resolution:** 2.10 Å<br>• **R-Value Free:** 0.224<br>• **R-Value Work:** 0.190<br>• **R-Value Observed:** 0.192

Metallothionein	Target Protein	1MHU	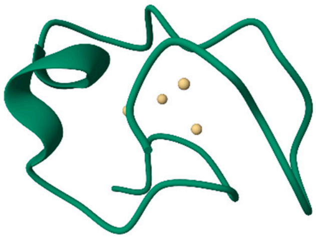	1MHU<br>*THE THREE-DIMENSIONAL STRUCTURE OF HUMAN [113CD7] METALLOTHIONEIN-2 IN SOLUTION DETERMINED BY NUCLEAR MAGNETIC RESONANCE SPECTROSCOPY*<br>• **PDB DOI:** https://doi.org/10.2210/pdb1MHU/pdb<br>• **BMRB:** 1154<br>• **Classification:** **METALLOTHIONEIN**<br>• **Organism(s):** Homo sapiens<br>• **Mutation(s):** No<br>• **Deposited:** 1990-05-14 **Released:** 1991-04-15<br>• **Deposition Author(s):** Braun, W., Messerle, B.A., Schaeffer, A., Vasak, M., Kaegi, J.H.R., Wuthrich, K.<br>**Experimental Data Snapshot**<br>• **Method:** SOLUTION NMR<br>• **Conformers Submitted:** 1
	Target Receptor	2MHU	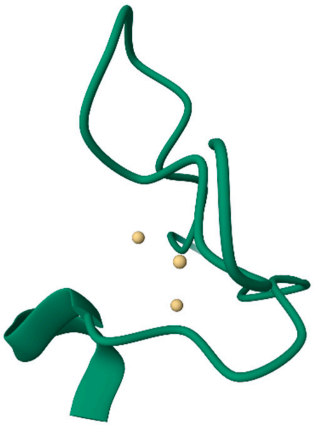	2MHU<br>*THE THREE-DIMENSIONAL STRUCTURE OF HUMAN [113CD7] METALLOTHIONEIN-2 IN SOLUTION DETERMINED BY NUCLEAR MAGNETIC RESONANCE SPECTROSCOPY*<br>• **PDB DOI:** https://doi.org/10.2210/pdb2MHU/pdb<br>• **Classification:** **METALLOTHIONEIN**<br>• **Organism(s):** Homo sapiens<br>• **Mutation(s):** No<br>• **Deposited:** 1990-05-14 **Released:** 1991-04-15<br>• **Deposition Author(s):** Braun, W., Messerle, B.A., Schaeffer, A., Vasak, M., Kaegi, J.H.R., Wuthrich, K.<br>**Experimental Data Snapshot**<br>• **Method:** SOLUTION NMR<br>• **Conformers Submitted:** 1

		2F5H	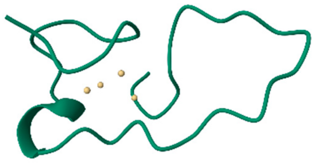	2F5H<br>*Solution structure of the alpha-domain of human Metallothionein-3*<br>• **PDB DOI:** https://doi.org/10.2210/pdb2F5H/pdb<br>• **Classification: METAL BINDING PROTEIN**<br>• **Organism(s):** Homo sapiens<br>• **Expression System:** Escherichia coli BL21<br>• **Mutation(s):** No<br>• **Deposited:** 2005-11-25 **Released:** 2006-05-30<br>• **Deposition Author(s):** Wang, H., Zhang, Q., Cai, B., Li, H.Y., Sze, K.H., Huang, Z.X., Wu, H.M., Sun, H.Z.<br>**Experimental Data Snapshot**<br>• **Method:** SOLUTION NMR<br>• **Conformers Calculated:** 30<br>• **Conformers Submitted:** 10<br>• **Selection Criteria:** best converged structures
Keratin	Target Protein	6EC0	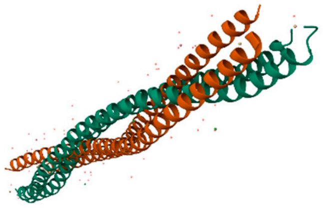	6EC0<br>*Crystal structure of the wild-type heterocomplex between coil 1B domains of human intermediate filament proteins keratin 1 (KRT1) and keratin 10 (KRT10)*<br>• **PDB DOI:** https://doi.org/10.2210/pdb6EC0/pdb<br>• **Classification: PROTEIN FIBRIL**<br>• **Organism(s):** Homo sapiens<br>• **Expression System:** Escherichia coli BL21(DE3)<br>• **Mutation(s):** No<br>• **Deposited:** 2018-08-07 **Released:** 2019-05-15<br>• **Deposition Author(s):** Eldirany, S.A., Lomakin, I.B., Bunick, C.G.<br>• **Funding Organization(s):** National Institutes of Health/National Institute of Arthritis and Musculoskeletal and Skin Diseases (NIH/NIAMS), Other private<br>**Experimental Data Snapshot**<br>• **Method:** X-RAY DIFFRACTION<br>• **Resolution:** 2.98 Å<br>• **R-Value Free:** 0.300<br>• **R-Value Work:** 0.281<br>• **R-Value Observed:** 0.282

	Target Receptor	4ZRY	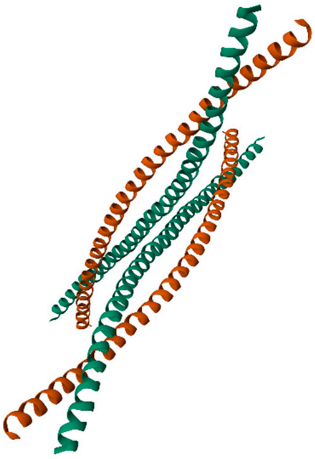	4ZRY<br>*Crystal structure of the heterocomplex between coil 2B domains of human intermediate filament proteins keratin 1 (KRT1) and keratin 10 (KRT10)*<br>• **PDB DOI:** https://doi.org/10.2210/pdb4ZRY/pdb<br>• **Classification: PROTEIN FIBRIL**<br>• **Organism(s):** Homo sapiens<br>• **Expression System:** Escherichia coli BL21(DE3)<br>• **Mutation(s):** No<br>• **Deposited:** 2015-05-12 **Released:** 2016-05-18<br>• **Deposition Author(s):** Bunick, C.G., Steitz, T.A.<br>• **Funding Organization(s):** Dermatology Foundation<br>**Experimental Data Snapshot**<br>• **Method:** X-RAY DIFFRACTION<br>• **Resolution:** 3.30 Å<br>• **R-Value Free:** 0.277<br>• **R-Value Work:** 0.273<br>• **R-Value Observed:** 0.273
Protein kinase enzyme	Target Protein	1P4F	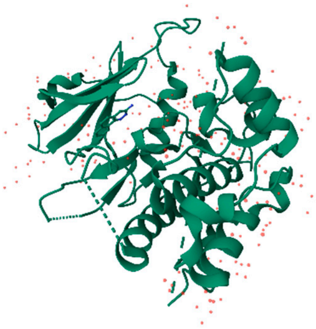	1P4F<br>*DEATH ASSOCIATED PROTEIN KINASE CATALYTIC DOMAIN WITH BOUND INHIBITOR FRAGMENT*<br>• **PDB DOI:** https://doi.org/10.2210/pdb1P4F/pdb<br>• **Classification: TRANSFERASE**<br>• **Organism(s):** Homo sapiens<br>• **Expression System:** Escherichia coli<br>• **Mutation(s):** No<br>• **Deposited:** 2003-04-23 **Released:** 2004-09-28<br>• **Deposition Author(s):** Velentza, A.V., Wainwright, M.S., Zasadzki, M., Mirzoeva, S., Haiech, J., Focia, P.J., Egli, M., Watterson, D.M.<br>**Experimental Data Snapshot**<br>• **Method:** X-RAY DIFFRACTION<br>• **Resolution:** 1.90 Å<br>• **R-Value Free:** 0.233<br>• **R-Value Work:** 0.185<br>• **R-Value Observed:** 0.186

		5IKP	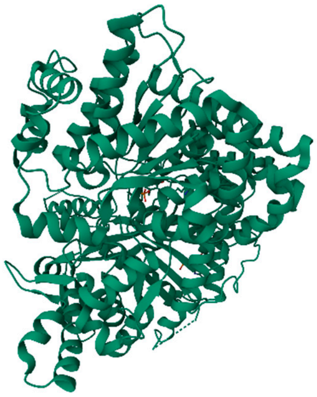	5IKP<br><br>*Crystal structure of human brain glycogen phosphorylase bound to AMP*<br><br>• **PDB DOI:** https://doi.org/10.2210/pdb5IKP/pdb<br>• **Classification:** **TRANSFERASE**<br>• **Organism(s):** Homo sapiens<br>• **Expression System:** Escherichia coli<br>• **Mutation(s):** No<br>• **Deposited:** 2016-03-03 **Released:** 2016-07-20<br>• **Deposition Author(s):** Mathieu, C., Li de la Sierra-Gallay, I., Xu, X., Haouz, A., Rodrigues-Lima, F.<br><br>**Experimental Data Snapshot**<br><br>• **Method:** X-RAY DIFFRACTION<br>• **Resolution:** 3.40 Å<br>• **R-Value Free:** 0.295<br>• **R-Value Work:** 0.202<br>• **R-Value Observed:** 0.207
	Target Receptor	1LHR	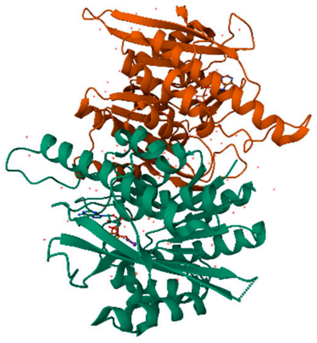	1LHR<br><br>*Crystal Structure of Pyridoxal Kinase complexed with ATP*<br><br>• **PDB DOI:** https://doi.org/10.2210/pdb1LHR/pdb<br>• **Classification:** **TRANSFERASE**<br>• **Organism(s):** Ovis aries<br>• **Mutation(s):** No<br>• **Deposited:** 2002-04-17 **Released:** 2003-02-11<br>• **Deposition Author(s):** Liang, D.C., Jiang, T., Li, M.H.<br><br>**Experimental Data Snapshot**<br><br>• **Method:** X-RAY DIFFRACTION<br>• **Resolution:** 2.60 Å<br>• **R-Value Free:** 0.222<br>• **R-Value Work:** 0.193<br>• **R-Value Observed:** 0.198

		6E0R	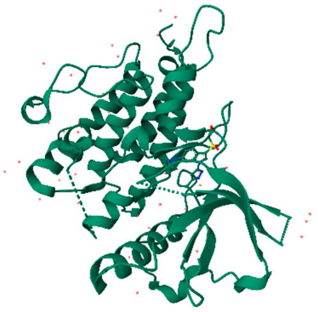	6E0R<br>*hALK in complex with compound 7 N-((1S)-1-(5-fluoropyridin-2-yl)ethyl)-1-(5-methyl-1H-pyrazol-3-yl)-3-(oxetan-3-ylsulfonyl)-1H-pyrrolo[2,3-b]pyridin-6-amine*<br>• **PDB DOI:** https://doi.org/10.2210/pdb6E0R/pdb<br>• **Classification: PROTEIN BINDING**<br>• **Organism(s):** Homo sapiens<br>• **Expression System:** unidentified baculovirus<br>• **Mutation(s):** No<br>• **Deposited:** 2018-07-06 **Released:** 2019-05-01<br>• **Deposition Author(s):** Lane, W., Saikatendu, K.<br>**Experimental Data Snapshot**<br>• **Method:** X-RAY DIFFRACTION<br>• **Resolution:** 2.30 Å<br>• **R-Value Free:** 0.296<br>• **R-Value Work:** 0.237<br>• **R-Value Observed:** 0.241
Beta Amyloid	Target Protein	5TXJ	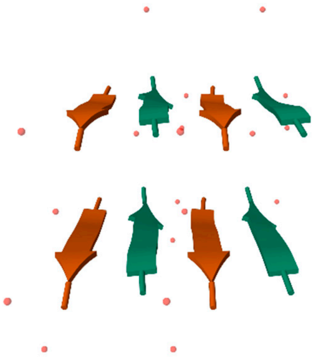	5TXJ<br>*Polymorphic form 1 of amyloid-beta derived peptide - IFAEDV*<br>• **PDB DOI:** https://doi.org/10.2210/pdb5TXJ/pdb<br>• **Classification: DE NOVO PROTEIN**<br>• **Organism(s):** Homo sapiens<br>• **Mutation(s):** No<br>• **Deposited:** 2016-11-16 **Released:** 2017-11-15<br>• **Deposition Author(s):** Sangwan, S., Sawaya, M.R., Eisenberg, D.<br>**Experimental Data Snapshot**<br>• **Method:** X-RAY DIFFRACTION<br>• **Resolution:** 1.13 Å<br>• **R-Value Free:** 0.157<br>• **R-Value Work:** 0.145<br>• **R-Value Observed:** 0.146

		3T4G	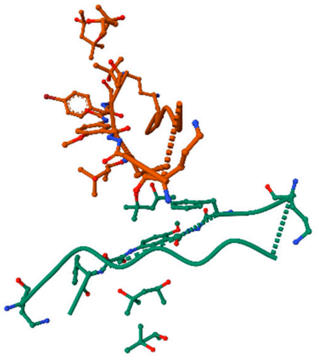	3T4G<br><br>*AIIGLMV segment from Alzheimer's Amyloid-Beta displayed on 54-membered macrocycle scaffold*<br><br>• **PDB DOI:** https://doi.org/10.2210/pdb3T4G/pdb<br>• **Classification:** **UNKNOWN FUNCTION**<br>• **Mutation(s):** No<br>• **Deposited:** 2011-07-26 **Released:** 2012-10-31<br>• **Deposition Author(s):** Zhao, M., Liu, C., Cheng, P.N., Eisenberg, D., Nowick, J.S.<br><br>**Experimental Data Snapshot**<br><br>• **Method:** X-RAY DIFFRACTION<br>• **Resolution:** 1.70 Å<br>• **R-Value Free:** 0.274<br>• **R-Value Work:** 0.236<br>• **R-Value Observed:** 0.240
	Target Receptor	3Q7G	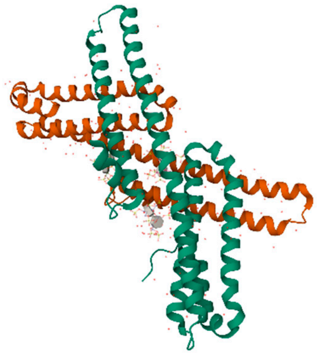	3Q7G<br><br>*Crystal Structure of E2 domain of Human Amyloid Precursor-Like Protein 1 in complex with SOS (sucrose octasulfate)*<br><br>• **PDB DOI:** https://doi.org/10.2210/pdb3Q7G/pdb<br>• **Classification:** **SIGNALING PROTEIN**<br>• **Organism(s):** Homo sapiens<br>• **Expression System:** Escherichia coli<br>• **Mutation(s):** No<br>• **Deposited:** 2011-01-04 **Released:** 2011-06-22<br>• **Deposition Author(s):** Xue, Y., Ha, Y.<br><br>**Experimental Data Snapshot**<br><br>• **Method:** X-RAY DIFFRACTION<br>• **Resolution:** 2.30 Å<br>• **R-Value Free:** 0.261<br>• **R-Value Work:** 0.217<br>• **R-Value Observed:** 0.222

ATPase	Target<br>Receptor	6WL<br>W	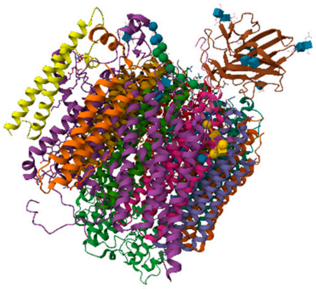	6WLW<br>*The Vo region of human V-ATPase in state 1 (focused refinement)*<br>• **PDB DOI:** https://doi.org/10.2210/pdb6WLW/pdb<br>• **EM Map EMD-21844:** EMDB EMDataResource<br>• **Classification:** **MEMBRANE PROTEIN**<br>• **Organism(s):** Homo sapiens<br>• **Mutation(s):** No<br>• **Membrane Protein:** Yes<br>• **Deposited:** 2020-04-20 **Released:** 2020-11-11<br>• **Deposition Author(s):** Wang, L., Wu, H., Fu, T.-M.<br>**Experimental Data Snapshot**<br>• **Method:** ELECTRON MICROSCOPY<br>• **Resolution:** 3.00 Å<br>• **Aggregation State:** PARTICLE<br>• **Reconstruction Method:** SINGLE PARTICLE
		6WM<br>3	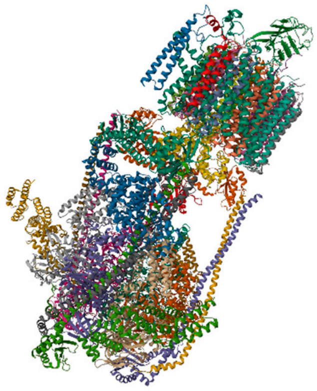	6WM3<br>*Human V-ATPase in state 2 with SidK and ADP*<br>• **PDB DOI:** https://doi.org/10.2210/pdb6WM3/pdb<br>• **EM Map EMD-21848:** EMDB EMDataResource<br>• **Classification:** **MEMBRANE PROTEIN**<br>• **Organism(s):** Homo sapiens, Legionella pneumophila subsp. pneumophila str. Philadelphia 1<br>• **Mutation(s):** No<br>• **Membrane Protein:** Yes<br>• **Deposited:** 2020-04-20 **Released:** 2020-11-11<br>• **Deposition Author(s):** Wang, L., Wu, H., Fu, T.M.<br>**Experimental Data Snapshot**<br>• **Method:** ELECTRON MICROSCOPY<br>• **Resolution:** 3.40 Å<br>• **Aggregation State:** PARTICLE<br>• **Reconstruction Method:** SINGLE PARTICLE

<table>
<tr>
<td rowspan="2">Albumin</td>
<td rowspan="2">Target Protein</td>
<td>5UJB</td>
<td>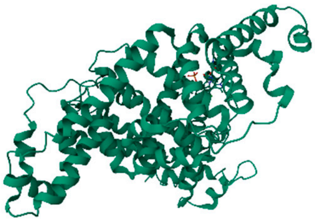</td>
<td>5UJB

Structure of a Mcl-1 Inhibitor Binding to Site 3 of Human Serum Albumin

• **PDB DOI:** https://doi.org/10.2210/pdb5UJB/pdb
• **Classification: TRANSPORT PROTEIN**
• **Organism(s):** Homo sapiens
• **Expression System:** Homo sapiens
• **Mutation(s):** No
• **Deposited:** 2017-01-17 **Released:** 2017-05-03
• **Deposition Author(s):** Zhao, B.
• **Funding Organization(s):** National Institutes of Health/National Cancer Institute (NIH/NCI)

Experimental Data Snapshot

• **Method:** X-RAY DIFFRACTION
• **Resolution:** 2.70 Å
• **R-Value Free:** 0.215
• **R-Value Work:** 0.174
• **R-Value Observed:** 0.177</td>
</tr>
<tr>
<td>6M5D</td>
<td>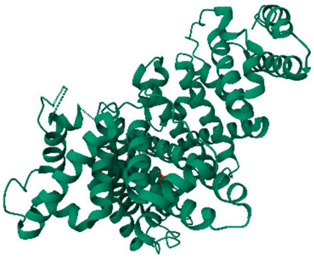</td>
<td>6M5D

Human serum albumin (apo form)

• **PDB DOI:** https://doi.org/10.2210/pdb6M5D/pdb
• **Classification: PEPTIDE BINDING PROTEIN**
• **Organism(s):** Homo sapiens
• **Expression System:** Homo sapiens
• **Mutation(s):** No
• **Deposited:** 2020-03-10 **Released:** 2020-11-18
• **Deposition Author(s):** Ito, S., Senoo, A., Nagatoishi, S., Yamamoto, M., Tsumoto, K., Wakui, N.
• **Funding Organization(s):** Japan Agency for Medical Research and Development (AMED)

Experimental Data Snapshot

• **Method:** X-RAY DIFFRACTION
• **Resolution:** 2.60 Å
• **R-Value Free:** 0.264
• **R-Value Work:** 0.230
• **R-Value Observed:** 0.232</td>
</tr>
</table>

	Target Receptor	6HSC	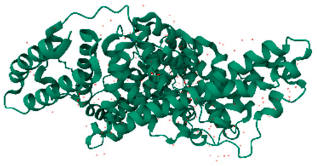	6HSC<br><br>*Structure of Human Serum Albumin in complex with Aristolochic Acid at 1.9 A resolution*<br><br>• **PDB DOI:** https://doi.org/10.2210/pdb6HSC/pdb<br>• **Classification:** **TRANSPORT PROTEIN**<br>• **Organism(s):** Homo sapiens<br>• **Expression System:** Homo sapiens<br>• **Mutation(s):** No<br>• **Deposited:** 2018-09-29 **Released:** 2019-10-23<br>• **Deposition Author(s):** Pomyalov, S., Sidorenko, V.S., Grollman, A.P., Shoham, G.<br><br>**Experimental Data Snapshot**<br><br>• **Method:** X-RAY DIFFRACTION<br>• **Resolution:** 1.90 Å<br>• **R-Value Free:** 0.286<br>• **R-Value Work:** 0.238<br>• **R-Value Observed:** 0.240
		2ESG	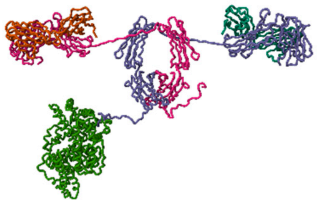	2ESG<br><br>*Solution structure of the complex between immunoglobulin IgA1 and human serum albumin*<br><br>• **PDB DOI:** https://doi.org/10.2210/pdb2ESG/pdb<br>• **Classification:** **IMMUNE SYSTEM/TRANSPORT PROTEIN**<br>• **Organism(s):** Homo sapiens<br>• **Expression System:** Escherichia coli<br>• **Mutation(s):** No<br>• **Deposited:** 2005-10-26 **Released:** 2006-01-31<br>• **Deposition Author(s):** Almogren, A., Furtado, P.B., Sun, Z., Perkins, S.J., Kerr, M.A.<br><br>**Experimental Data Snapshot**<br><br>• **Method:** SOLUTION SCATTERING

		6YG9	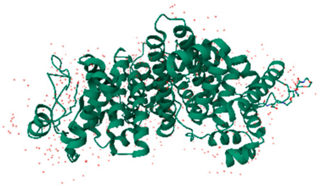	6YG9<br>*CRYSTAL STRUCTURE OF HUMAN SERUM ALBUMIN (HSA) IN COMPLEX WITH GN-07.*<br>• **PDB DOI:** https://doi.org/10.2210/pdb6YG9/pdb<br>• **Classification: TRANSPORT PROTEIN**<br>• **Organism(s):** Homo sapiens<br>• **Mutation(s):** No<br>• **Deposited:** 2020-03-27 **Released:** 2021-01-13<br>• **Deposition Author(s):** Schreuder, H.A., Liesum, A.<br>**Experimental Data Snapshot**<br>• **Method:** X-RAY DIFFRACTION<br>• **Resolution:** 1.89 Å<br>• **R-Value Free:** 0.313<br>• **R-Value Work:** 0.239<br>• **R-Value Observed:** 0.243
Mono amino oxidase (MAO)	Target Protein	2BK3	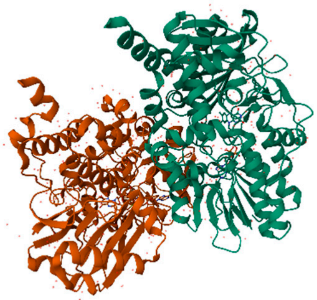	2BK3<br>*Human Monoamine Oxidase B in complex with Farnesol*<br>• **PDB DOI:** https://doi.org/10.2210/pdb2BK3/pdb<br>• **Classification: OXIDOREDUCTASE**<br>• **Organism(s):** Homo sapiens<br>• **Expression System:** Komagataella pastoris<br>• **Mutation(s):** No<br>• **Membrane Protein:** Yes<br>• **Deposited:** 2005-02-10 **Released:** 2005-02-14<br>• **Deposition Author(s):** Binda, C., Edmondson, D.E., Mattevi, A.<br>**Experimental Data Snapshot**<br>• **Method:** X-RAY DIFFRACTION<br>• **Resolution:** 1.80 Å<br>• **R-Value Free:** 0.249<br>• **R-Value Work:** 0.225<br>• **R-Value Observed:** 0.226

		2BXS	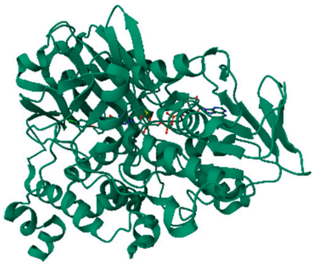	2BXS<br>*Human Monoamine Oxidase A in complex with Clorgyline, Crystal Form B*<br>• **PDB DOI:** https://doi.org/10.2210/pdb2BXS/pdb<br>• **Classification: OXIDOREDUCTASE**<br>• **Organism(s):** Homo sapiens<br>• **Expression System:** Komagataella pastoris<br>• **Mutation(s):** No<br>• **Membrane Protein:** Yes<br>• **Deposited:** 2005-07-27 **Released:** 2005-08-09<br>• **Deposition Author(s):** De Colibus, L., Binda, C., Edmondson, D.E., Mattevi, A.<br>**Experimental Data Snapshot**<br>• **Method:** X-RAY DIFFRACTION<br>• **Resolution:** 3.15 Å<br>• **R-Value Free:** 0.330<br>• **R-Value Work:** 0.268<br>• **R-Value Observed:** 0.271
		7DJU	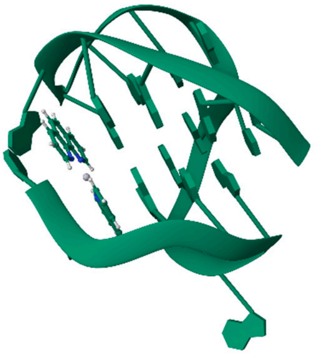	7DJU<br>*NMR solution structure of the 1:1 complex of a platinum(II) compound bound to 5'-end of Myc1234 G-quadruplex reveals the mechanism of conformational switch and dynamic binding of G-quadruplex*<br>• **PDB DOI:** https://doi.org/10.2210/pdb7DJU/pdb<br>• **NDB:** 7DJU<br>• **Classification: DNA**<br>• **Organism(s):** Homo sapiens<br>• **Mutation(s):** No<br>• **Deposited:** 2020-11-22 **Released:** 2022-01-12<br>• **Deposition Author(s):** Liu, W., Mao, Z.W.<br>• **Funding Organization(s):** National Natural Science Foundation of China (NSFC), Ministry of Education (MoE, China)<br>**Experimental Data Snapshot**<br>• **Method:** SOLUTION NMR<br>• **Conformers Calculated:** 100<br>• **Conformers Submitted:** 20<br>• **Selection Criteria:** structures with the lowest energy

	Target Receptor	7EL7	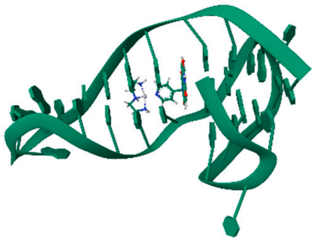	7EL7<br>*NMR solution structure of the 1:1 complex of a quadruplex-duplex hybrid MYT1L and a platinum(II) ligand L1Pt(dien)*<br>• **PDB DOI:** https://doi.org/10.2210/pdb7EL7/pdb<br>• **NDB:** 7EL7<br>• **Classification:** **DNA**<br>• **Organism(s):** Homo sapiens<br>• **Mutation(s):** No<br>• **Deposited:** 2021-04-08 **Released:** 2021-12-15<br>• **Deposition Author(s):** Liu, L.-Y., Liu, W., Mao, Z.-W.<br>• **Funding Organization(s):** National Science Foundation (NSF, China)<br>**Experimental Data Snapshot**<br>• **Method:** SOLUTION NMR<br>• **Conformers Calculated:** 100<br>• **Conformers Submitted:** 15<br>• **Selection Criteria:** structures with the lowest energy
		2VRM	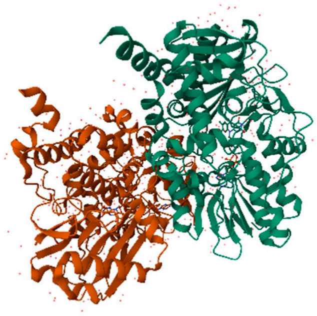	2VRM<br>*Structure of human MAO B in complex with phenyethylhydrazine*<br>• **PDB DOI:** https://doi.org/10.2210/pdb2VRM/pdb<br>• **Classification:** **OXIDOREDUCTASE**<br>• **Organism(s):** Homo sapiens<br>• **Expression System:** Komagataella pastoris<br>• **Mutation(s):** No<br>• **Membrane Protein:** Yes<br>• **Deposited:** 2008-04-09 **Released:** 2008-04-22<br>• **Deposition Author(s):** Binda, C., Wang, J., Li, M., Hubalek, F., Mattevi, A., Edmondson, D.E.<br>**Experimental Data Snapshot**<br>• **Method:** X-RAY DIFFRACTION<br>• **Resolution:** 2.30 Å<br>• **R-Value Free:** 0.246<br>• **R-Value Work:** 0.200<br>• **R-Value Observed:** 0.201

adrenaline	Target Protein	2HKK	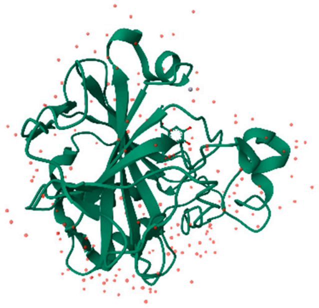	2HKK<br><br>*Carbonic anhydrase activators: Solution and X-ray crystallography for the interaction of andrenaline with various carbonic anhydrase isoforms*<br><br>• **PDB DOI:** https://doi.org/10.2210/pdb2HKK/pdb<br>• **Classification: LYASE**<br>• **Organism(s):** Homo sapiens<br>• **Mutation(s):** No<br>• **Deposited:** 2006-07-05 **Released:** 2007-05-22<br>• **Deposition Author(s):** Temperini, C., Innocenti, A., Vullo, D., Scozzafava, A., Supuran, C.T.<br><br>**Experimental Data Snapshot**<br><br>• **Method:** X-RAY DIFFRACTION<br>• **Resolution:** 1.90 Å<br>• **R-Value Free:** 0.233<br>• **R-Value Work:** 0.186<br>• **R-Value Observed:** 0.188
		7BTS	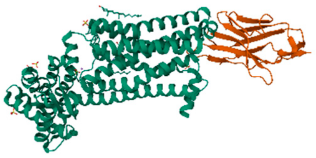	7BTS<br><br>*Structure of human beta1 adrenergic receptor bound to epinephrine and nanobody 6B9*<br><br>• **PDB DOI:** https://doi.org/10.2210/pdb7BTS/pdb<br>• **Classification: MEMBRANE PROTEIN**<br>• **Organism(s):** Tequatrovirus T4, Homo sapiens, Vicugna pacos<br>• **Expression System:** Spodoptera frugiperda<br>• **Mutation(s):** Yes<br>• **Membrane Protein:** Yes<br>• **Deposited:** 2020-04-02 **Released:** 2020-12-02<br>• **Deposition Author(s):** Xu, X., Kaindl, J., Clark, M., Hubner, H., Hirata, K., Sunahara, R., Gmeiner, P., Kobilka, B.K., Liu, X.<br><br>**Experimental Data Snapshot**<br><br>• **Method:** X-RAY DIFFRACTION<br>• **Resolution:** 3.13 Å<br>• **R-Value Free:** 0.259<br>• **R-Value Work:** 0.235<br>• **R-Value Observed:** 0.237

	Target Receptor	2RH1	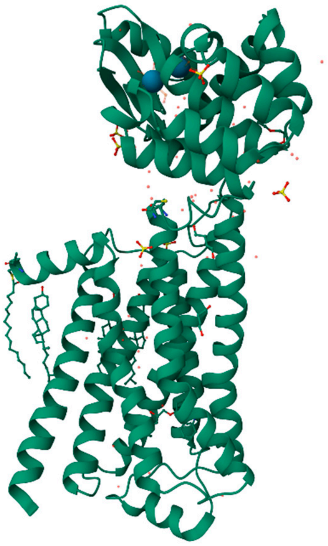	2RH1<br>*High resolution crystal structure of human B2-adrenergic G protein-coupled receptor.*<br>• **PDB DOI:** https://doi.org/10.2210/pdb2RH1/pdb<br>• **Classification:** **MEMBRANE PROTEIN / HYDROLASE**<br>• **Organism(s):** Homo sapiens, Tequatrovirus T4<br>• **Expression System:** Spodoptera frugiperda<br>• **Mutation(s):** Yes<br>• **Membrane Protein:** Yes<br>• **Deposited:** 2007-10-05 **Released:** 2007-10-30<br>• **Deposition Author(s):** Cherezov, V., Rosenbaum, D.M., Hanson, M.A., Rasmussen, S.G.F., Thian, F.S., Kobilka, T.S., Choi, H.J., Kuhn, P., Weis, W.I., Kobilka, B.K., Stevens, R.C., Accelerated Technologies Center for Gene to 3D Structure (ATCG3D), GPCR Network (GPCR)<br>**Experimental Data Snapshot**<br>• **Method:** X-RAY DIFFRACTION<br>• **Resolution:** 2.40 Å<br>• **R-Value Free:** 0.232<br>• **R-Value Work:** 0.196<br>• **R-Value Observed:** 0.198
Cortisol	Target Protein	2VDX	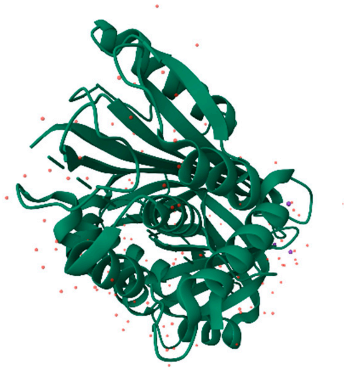	2VDX<br>*Crystal Structure of the reactive loop Cleaved Corticosteroid Binding Globulin*<br>• **PDB DOI:** https://doi.org/10.2210/pdb2VDX/pdb<br>• **Classification:** **TRANSPORT PROTEIN**<br>• **Organism(s):** Homo sapiens<br>• **Expression System:** Escherichia coli BL21(DE3)<br>• **Mutation(s):** No<br>• **Deposited:** 2007-10-13 **Released:** 2008-05-06<br>• **Deposition Author(s):** Zhou, A., Wei, Z., Read, R.J.<br>**Experimental Data Snapshot**<br>• **Method:** X-RAY DIFFRACTION<br>• **Resolution:** 1.84 Å<br>• **R-Value Free:** 0.241<br>• **R-Value Work:** 0.192<br>• **R-Value Observed:** 0.194

		2VDY	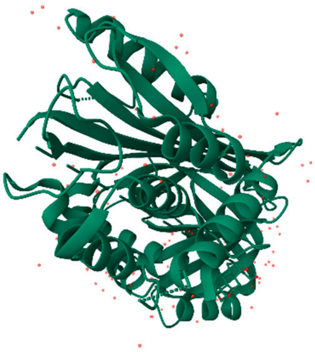	2VDY<br><br>*Crystal structure of the reactive loop cleaved Corticosteroid Binding Globulin complexed with Cortisol*<br><br>• **PDB DOI:** https://doi.org/10.2210/pdb2VDY/pdb<br>• **Classification: TRANSPORT PROTEIN**<br>• **Organism(s):** Homo sapiens<br>• **Expression System:** Escherichia coli BL21(DE3)<br>• **Mutation(s):** No<br>• **Deposited:** 2007-10-13 **Released:** 2008-05-13<br>• **Deposition Author(s):** Zhou, A., Wei, Z., Read, R.J.<br><br>**Experimental Data Snapshot**<br><br>• **Method:** X-RAY DIFFRACTION<br>• **Resolution:** 2.30 Å<br>• **R-Value Free:** 0.271<br>• **R-Value Work:** 0.210<br>• **R-Value Observed:** 0.213
	Target Receptor	4P6X	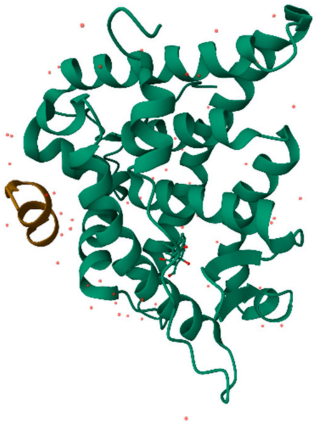	4P6X<br><br>*Crystal Structure of cortisol-bound glucocorticoid receptor ligand binding domain*<br><br>• **PDB DOI:** https://doi.org/10.2210/pdb4P6X/pdb<br>• **Classification: HORMONE RECEPTOR/HORMONE ACTIVATOR**<br>• **Organism(s):** Homo sapiens<br>• **Expression System:** Escherichia coli K-12<br>• **Mutation(s):** Yes<br>• **Deposited:** 2014-03-25 **Released:** 2014-04-16<br>• **Deposition Author(s):** He, Y., Zhou, X.E., Tolbert, W.D., Powell, K., Melcher, K., Xu, H.E.<br>• **Funding Organization(s):** National Institutes of Health/National Institute of Diabetes and Digestive and Kidney Disease (NIH/NIDDK), AAF<br><br>**Experimental Data Snapshot**<br><br>• **Method:** X-RAY DIFFRACTION<br>• **Resolution:** 2.50 Å<br>• **R-Value Free:** 0.276<br>• **R-Value Work:** 0.246<br>• **R-Value Observed:** 0.248

TNF-α	Target<br>Cytokine	6RMJ	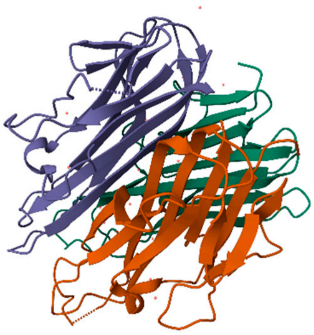	6RMJ<br>*Crystal structure of human NGR-TNF*<br>• **PDB DOI:** https://doi.org/10.2210/pdb6RMJ/pdb<br>• **Classification:** **CYTOKINE**<br>• **Organism(s):** Homo sapiens<br>• **Expression System:** Escherichia coli BL21(DE3)<br>• **Mutation(s):** No<br>• **Deposited:** 2019-05-07 **Released:** 2019-10-09<br>• **Deposition Author(s):** Degano, M., Garau, G.<br>**Experimental Data Snapshot**<br>• **Method:** X-RAY DIFFRACTION<br>• **Resolution:** 2.65 Å<br>• **R-Value Free:** 0.253<br>• **R-Value Work:** 0.209<br>• **R-Value Observed:** 0.211
	Target<br>Receptor	5TLJ	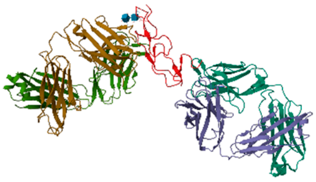	5TLJ<br>*COMPLEX BETWEEN HUMAN CD27 AND FAB FRAGMENTS OF ANTIBODIES M2177 AND M2191*<br>• **PDB DOI:** https://doi.org/10.2210/pdb5TLJ/pdb<br>• **Classification:** **IMMUNE SYSTEM**<br>• **Organism(s):** Mus musculus, Homo sapiens<br>• **Expression System:** Homo sapiens, unidentified baculovirus<br>• **Mutation(s):** No<br>• **Deposited:** 2016-10-11 **Released:** 2017-02-08<br>• **Deposition Author(s):** Teplyakov, A., Obmolova, G., Malia, T., Gilliland, G.L.<br>**Experimental Data Snapshot**<br>• **Method:** X-RAY DIFFRACTION<br>• **Resolution:** 3.50 Å<br>• **R-Value Free:** 0.356<br>• **R-Value Work:** 0.283<br>• **R-Value Observed:** 0.287

		6PE7	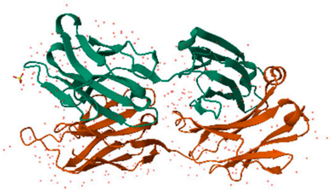	6PE7<br>*Crystal Structure of ABBV-323 FAB*<br>• **PDB DOI:** https://doi.org/10.2210/pdb6PE7/pdb<br>• **Classification: IMMUNE SYSTEM**<br>• **Organism(s):** Homo sapiens<br>• **Expression System:** Homo sapiens<br>• **Mutation(s):** No<br>• **Deposited:** 2019-06-20 **Released:** 2019-08-14<br>• **Deposition Author(s):** Argiriadi, M.A.<br>**Experimental Data Snapshot**<br>• **Method:** X-RAY DIFFRACTION<br>• **Resolution:** 1.74 Å<br>• **R-Value Free:** 0.230<br>• **R-Value Work:** 0.196<br>• **R-Value Observed:** 0.198
IL-1β	Target Receptor	4GAF	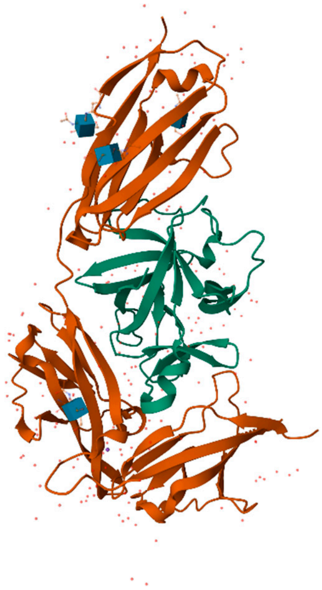	4GAF<br>*Crystal structure of EBI-005, a chimera of human IL-1beta and IL-1Ra, bound to human Interleukin-1 receptor type 1*<br>• **PDB DOI:** https://doi.org/10.2210/pdb4GAF/pdb<br>• **Classification: SIGNALING PROTEIN**<br>• **Organism(s):** Homo sapiens<br>• **Expression System:** Escherichia coli<br>• **Mutation(s):** No<br>• **Deposited:** 2012-07-25 **Released:** 2013-02-20<br>• **Deposition Author(s):** Hou, J., Townson, S.A., Kovalchin, J.T., Masci, A., Kiner, O., Shu, Y., King, B., Thomas, C., Garcia, K.C., Furfine, E.S., Barnes, T.M.<br>**Experimental Data Snapshot**<br>• **Method:** X-RAY DIFFRACTION<br>• **Resolution:** 2.15 Å<br>• **R-Value Free:** 0.271<br>• **R-Value Work:** 0.215<br>• **R-Value Observed:** 0.218

		4GAI	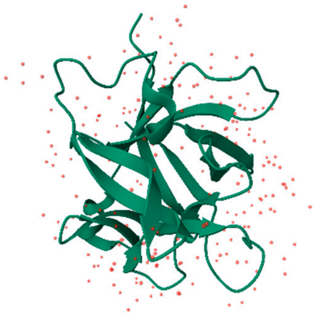	4GAI<br>*Crystal structure of EBI-005, a chimera of human IL-1beta and IL-1Ra*<br>• **PDB DOI:** https://doi.org/10.2210/pdb4GAI/pdb<br>• **Classification:** **SIGNALING PROTEIN**<br>• **Organism(s):** Homo sapiens<br>• **Expression System:** Escherichia coli<br>• **Mutation(s):** No<br>• **Deposited:** 2012-07-25 **Released:** 2013-02-20<br>• **Deposition Author(s):** Hou, J., Townson, S.A., Kovalchin, J.T., Masci, A., Kiner, O., Shu, Y., King, B., Thomas, C., Garcia, K.C., Furfine, E.S., Barnes, T.M.<br>**Experimental Data Snapshot**<br>• **Method:** X-RAY DIFFRACTION<br>• **Resolution:** 1.49 Å<br>• **R-Value Free:** 0.219<br>• **R-Value Work:** 0.187<br>• **R-Value Observed:** 0.189
COX-2	Target Protein	5JW1	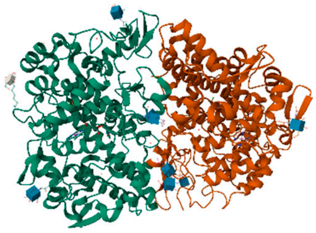	5JW1<br>*Crystal structure of Celecoxib bound to S121P murine COX-2 mutant*<br>• **PDB DOI:** https://doi.org/10.2210/pdb5JW1/pdb<br>• **Classification:** **OXIDOREDUCTASE**<br>• **Organism(s):** Mus musculus<br>• **Expression System:** Spodoptera frugiperda<br>• **Mutation(s):** Yes<br>• **Deposited:** 2016-05-11 **Released:** 2016-10-26<br>• **Deposition Author(s):** Malkowski, M.G., Orlando, B.J.<br>**Experimental Data Snapshot**<br>• **Method:** X-RAY DIFFRACTION<br>• **Resolution:** 2.82 Å<br>• **R-Value Free:** 0.226<br>• **R-Value Work:** 0.189<br>• **R-Value Observed:** 0.191

	Target Receptor	4E1G	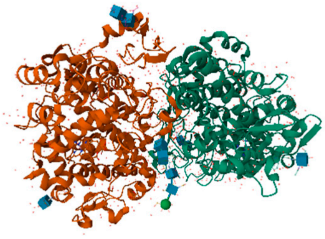	4E1G<br>*X-ray crystal structure of alpha-linolenic acid bound to the cyclooxygenase channel of cyclooxygenase-2*<br>• **PDB DOI:** https://doi.org/10.2210/pdb4E1G/pdb<br>• **Classification: OXIDOREDUCTASE**<br>• **Organism(s):** Mus musculus<br>• **Expression System:** Spodoptera frugiperda<br>• **Mutation(s):** Yes<br>• **Deposited:** 2012-03-06 **Released:** 2012-04-25<br>• **Deposition Author(s):** Vecchio, A.J., Malkowski, M.G.<br>**Experimental Data Snapshot**<br>• **Method:** X-RAY DIFFRACTION<br>• **Resolution:** 2.10 Å<br>• **R-Value Free:** 0.203<br>• **R-Value Work:** 0.159<br>• **R-Value Observed:** 0.161
		3TZI	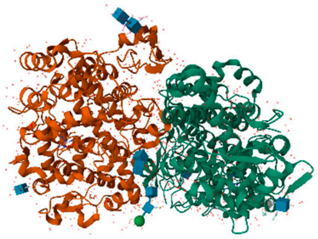	3TZI<br>*X-ray crystal structure of arachidonic acid bound in the cyclooxygenase channel of G533V murine COX-2*<br>• **PDB DOI:** https://doi.org/10.2210/pdb3TZI/pdb<br>• **Classification: OXIDOREDUCTASE**<br>• **Organism(s):** Mus musculus<br>• **Expression System:** Spodoptera frugiperda<br>• **Mutation(s):** Yes<br>• **Deposited:** 2011-09-27 **Released:** 2012-04-25<br>• **Deposition Author(s):** Vecchio, A.J., Malkowski, M.G.<br>**Experimental Data Snapshot**<br>• **Method:** X-RAY DIFFRACTION<br>• **Resolution:** 2.15 Å<br>• **R-Value Free:** 0.210<br>• **R-Value Work:** 0.170<br>• **R-Value Observed:** 0.172

<table>
<tr>
<td rowspan="2">LOX</td>
<td rowspan="2">Target
Receptor</td>
<td>1YPO</td>
<td>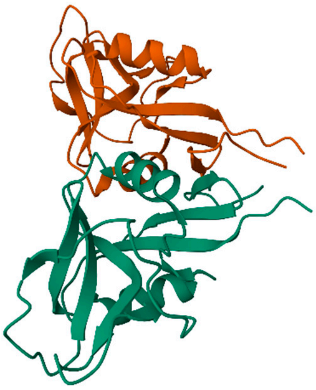</td>
<td>1YPO

Human Oxidized Low Density Lipoprotein Receptor LOX-1 P3 1 21 Space Group

• PDB DOI: https://doi.org/10.2210/pdb1YPO/pdb
• Classification: IMMUNE SYSTEM
• Organism(s): Homo sapiens
• Expression System: Escherichia coli
• Mutation(s): No
• Deposited: 2005-01-31 Released: 2005-02-08
• Deposition Author(s): Park, H., Adsit, F.G., Boyington, J.C.

Experimental Data Snapshot

• Method: X-RAY DIFFRACTION
• Resolution: 3.00 Å
• R-Value Free: 0.285
• R-Value Work: 0.228
• R-Value Observed: 0.228</td>
</tr>
<tr>
<td>1YPU</td>
<td>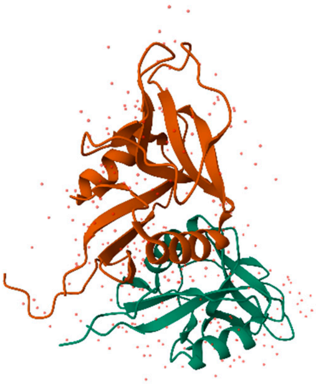</td>
<td>1YPU

Human Oxidized Low Density Lipoprotein Receptor LOX-1 C2 Space Group

• PDB DOI: https://doi.org/10.2210/pdb1YPU/pdb
• Classification: IMMUNE SYSTEM
• Organism(s): Homo sapiens
• Expression System: Escherichia coli
• Mutation(s): No
• Deposited: 2005-01-31 Released: 2005-02-08
• Deposition Author(s): Park, H., Adsit, F.G., Boyington, J.C.

Experimental Data Snapshot

• Method: X-RAY DIFFRACTION
• Resolution: 2.05 Å
• R-Value Free: 0.238
• R-Value Work: 0.183
• R-Value Observed: 0.183</td>
</tr>
</table>
